# Botulinum Toxin A for the Treatment of a Child with SUNCT Syndrome

**DOI:** 10.1155/2016/8016065

**Published:** 2016-04-06

**Authors:** Yi Zhang, Haifeng Zhang, Ya-Jun Lian, Yun-Qing Ma, Nan-Chang Xie, Xuan Cheng, Lu Zhang

**Affiliations:** Department of Neurology, The First Affiliated Hospital, Zhengzhou University, Zhengzhou 450000, China

## Abstract

*Background.* Short-lasting unilateral neuralgiform headache with conjunctival injection and tearing (SUNCT) syndrome is an unusual cause of headache, mainly described in older adults, and is rare in children. Pain attacks may be severe, frequent, and prolonged. The therapeutic benefits of many drugs are disappointing.* Patient and Methods.* A 12-year-old boy suffered severe headache and toothache for 20 days. As treatment with nonsteroidal anti-inflammatory drugs, anticonvulsants, and steroids proved ineffective, he was treated with ipsilateral multisite subcutaneous injections of botulinum toxin A 70 U around the orbit, the temporal area, and the upper gum.* Results.* The pain had reduced in frequency and severity by the fourth day after treatment and had completely disappeared after 7 days. There were no side effects or recurrence during a subsequent 17-month follow-up period.* Conclusion.* Botulinum toxin A can be used to treat the first episode of SUNCT in children over the age of 12 years.

## 1. Introduction

Short-lasting unilateral neuralgiform headache with conjunctival injection and tearing (SUNCT) syndrome is an unusual cause of headache and is mainly found in middle aged or older patients. It is rare in children. The clinical features of SUNCT syndrome are a transient onset of severe unilateral orbital pain, accompanied by ipsilateral conjunctival injection and tearing. It is classified, along with cluster headache and paroxysmal hemicranias, as a trigeminal autonomic cephalalgia (TAC). SUNCT syndrome may be difficult to diagnose and challenging to treat. Recently, gabapentin, lamotrigine, oxcarbazepine, and topiramate have been reported to have some therapeutic effects, but the extent of the therapeutic benefits of anticonvulsants and nonsteroidal anti-inflammatory drugs (NSAIDs) is unsatisfactory in many cases. Here, we describe the successful use of botulinum toxin A in the treatment of SUNCT syndrome in a 12-year-old boy, in whom a number of standard drug treatments had proved ineffective.

## 2. Patient and Methods

The patient, a 12-year-old boy, was admitted to the Pediatric Department of our hospital in April 2013 with a 20-day history of severe paroxysmal unilateral headache and toothache. The weight of the patient is 50 kg. He complained of spontaneous pain in the left orbital and temporal areas, accompanied by severe pain in the left upper tooth (rated 9 out of 10 on a visual analog scale, VAS). He experienced 10 pain attacks each day that resolved spontaneously after about 1 minute, which mainly occurred during daytime and were accompanied by redness and tearing of the ipsilateral eye, a running nose, and left-sided facial flushing. Clinical examination revealed left facial hyperalgesia but no other abnormality. Cranial magnetic resonance imaging (MRI), angiography, and venography were normal. Lumbar puncture revealed normal cerebrospinal fluid pressure and biochemical and cytological profiles. Routine blood tests were normal, as were the serum C-reactive protein concentration, erythrocyte sedimentation rate, and an autoimmune screen.

Oral carbamazepine 200 mg three times a day was commenced, but there was no change in the severity or frequency of pain attacks. The patient had allergic reaction after four-day use. Allergic symptoms include sample urticaria rash on his face and both legs. The rash was itchy and faded when pressed. Thus, carbamazepine was discontinued immediately. After transfer to our department, gabapentin 100 mg was administered orally three times a day (the reason to use the low dose was to prevent side effects), without relief. Similarly, pregabalin (75 mg twice daily), indomethacin (25 mg three times daily), flunarizine (5 mg at night), ibuprofen (300 mg four times daily), topiramate (25 mg twice daily, gradually increased to 150 mg/d), and methylprednisolone (80 mg intravenously once daily) were used, 7–10 L/min of pure oxygen was used for 10–20 min per day, and 2% of lidocaine 2 mL nasal drops (once daily) was trialed for 4 days, without obvious improvement. The drugs were changed in a short time because they could not relieve the pain and the patient's parents urged us to change drugs. In this situation, we considered trying botulinum toxin A treatment. We had given a clear and comprehensive description of the indication, the usage, and the risks of the botulinum toxin type A to the child and his parents prior to medication. And the parents signed the informed consent. With the agreement of his parents, botulinum toxin A treatment was given. Botulinum toxin A (100 U, diluted in 2 mL 0.9% NaCl) was injected subcutaneously in the ipsilateral upper gum, temporal area, orbit, and cheek, at a dose of 2.5–5 U at each site, separated by 1.5 cm. After 4 days, the pain began to improve and attacks became less frequent. After 7 days, the pain had completely resolved and the patient was discharged, having stopped all medication. Seventeen months after the initial episode, there has been no recurrence of symptoms.

## 3. Discussion

SUNCT syndrome is an unusual form of headache typified by severe, transient, unilateral, orbital pain attacks, accompanied by ipsilateral conjunctival injection and tearing. Sjaastad and colleagues reported in 1989 [1]. The International Headache Society proposed the following set of diagnostic criteria in 2004: needle-prick-like or pulsatile headache attacks in a unilateral orbital, upper orbital, or temporal area; duration of 5–240 s accompanied by ipsilateral conjunctival injection and tearing; frequency of three to 200 attacks per day; and exclusion of other diagnoses [2]. It is most often found in middle aged or older patients; the mean age at presentation is 50 years. The youngest patient to be diagnosed with SUNCT reported in the literature was 5 years old [3]; the oldest was 88 years old [4]. The onset of SUNCT is influenced by the seasons, as it presents most frequently in the spring and autumn [5]. SUNCT is usually sporadic, but familial disease has been reported [6].

The cause of SUNCT syndrome is usually not identified: the pathogenesis and pathophysiology are unclear. Functional MRI has identified active metabolism and increased perfusion in the hypothalamus [3], suggesting that SUNCT may be provoked by abnormal activity in the hypothalamus, in turn brought on after the activation of the trigeminocervical complex and other central nervous system autonomic nerves [5]. Secondary SUNCT syndrome is less common [7], but it can arise as a result of abnormalities of vessels at the cerebellopontine angle, cerebellopontine angle venous angioma, brainstem angiocavernoma, eye trauma, a complication of AIDS infection in the posterior cranial fossa, direct compression of the trigeminal nerve by cerebral vessels, lesions of the trigeminal nerve, compression of the brainstem (e.g., in osteogenesis imperfecta), dorsolateral brainstem infarction, HIV infection, prolactin-secreting pituitary adenoma, cerebellopontine angle astrocytoma, leiomyosarcoma of the venous sinus, extracranial intraorbital cystic tumor, neurofibromatosis type 2, intracranial intraorbital metastasis, or invasion of the cavernous sinus by macroprolactinoma [8]. It has been reported that treatment of prolactinoma with dopamine 2 receptor agonists such as bromocriptine, lisuride, cabergoline, and quinagolide may provoke the disease [9].

SUNCT presents with pain in and around the orbit, often radiating to the ipsilateral forehead, temporal area, nose, cheek, or upper mandible. The pain is severe and unbearable and may be burning, sharp, or pulsatile in nature. It begins quickly, peaking within 2-3 s, persists for several seconds to minutes, and has no signs. The second edition of the International Headache Society classification [2] defines the pain onset as occurring within 5–240 s, with 3–200 attacks per day, but the recently revised 2013 edition states that onset may occur over 1 s to 10 minutes and that there may be two or more attacks per day [10]. In contrast with trigeminal neuralgia, SUNCT has no refractory period [11]. The pain of SUNCT is accompanied by conjunctival injection, tearing, and other autonomic nerve signs. Favoni and colleagues [12] examined autonomic changes in 158 patients with SUNCT syndrome and found that 100% had conjunctival injection and tearing, while 48.7% had mucus discharge from the nose, 32.9% nasal congestion, 31.6% eyelid edema, 29.7% ptosis, 7.6% facial flushing, 7.6% facial sweating, 4.4% myosis, 1.3% mydriasis, and 0.6% salivation, while 2.5% had other miscellaneous signs. Triggers included touching of the orbital, temporal, eye, or nasal areas, chewing, the wind, bright light, loud noise, and heat. Blowing the nose, sneezing, or gently massaging the painful area on the head or face may ameliorate the pain. Alcohol is not a trigger. Among TACs, SUNCT should be discriminated from cluster headache, paroxysmal hemicranias [13], and trigeminal neuralgia [4].

The treatment of SUNCT syndrome is challenging; drugs tend to be the first choice. As SUNCT is characteristic of neuropathic pain, anticonvulsants are often used; however, the evidence for their use is not underpinned by any randomized controlled trials. Favoni and colleagues also summarized the drugs used successfully in their cohort of patients: lamotrigine 400 mg per day was effective in 52 out of 81 patients (64.2%), gabapentin 3,600 mg per day in 24 out of 71 (33.8%), topiramate in 14 out of 43 (32.6%), carbamazepine 200–1,200 mg per day in 25 out of 119 (21%), local nerve block with lidocaine in nine out of 11 (81.8%), and intravenous lidocaine in 22 out of 26 patients (84.6%) [12]. Oxcarbazepine was effective in 14.3% of patients for whom it was prescribed, zonisamide for 50%, a corticosteroid for 31.8%, verapamil for 5.5%, and indomethacin for 0.78% [12].

For patients for whom drug therapy has proved ineffective, more invasive techniques such as microvascular decompression, local nerve block, glycerol, gamma knife or radiofrequency ablation, percutaneous balloon compression, and nerve stimulation may be employed. In one study, 75% of the patients (12 out of 16) undergoing microvascular decompression for SUNCT had achieved complete relief from symptoms within 32 months of surgery [12]. Greater occipital nerve block has also proved effective, but in a smaller proportion of patients: in two studies seven out of 25 patients (25%) reported favourable outcomes after nerve block with lidocaine and methylprednisolone (five cases), bupivacaine (one case), and triamcinolone acetonide, lidocaine, and dexamethasone (one case) [14, 15]. Three patients with SUNCT have reported complete relief from symptoms after glycerol ablation, lasting from several weeks to 4 years [16]. Of the two cases treated with gamma knife ablation of the trigeminal and sphenopalatine ganglia, one achieved complete pain relief for 39 months without the need for analgesics [17], while the other had intermittent symptoms during 4 months of initial follow-up [18]. One of the two patients treated with gamma knife ablation of the trigeminal ganglion reported improved pain relief for 2 months and the other experienced no benefit [19], while the two patients who underwent radiofrequency thermocoagulation of the trigeminal ganglia experienced 2 and 3 years of pain relief [20, 21]. One of the two patients treated with deep brain stimulation reported a benefit without the need for additional drugs [22], while the other still required regular lamotrigine [23].

Our patient's symptoms met the diagnostic criteria of the International Headache Society [2], but showing no response to a panel of drugs. The literature contains one report of the use of botulinum toxin A in the treatment of SUNCT in a 55-year-old man with a 20-year history of symptoms refractory to multiple drugs, in whom botulinum toxin A 40 U was injected subcutaneously around the orbit on the affected side, shared between four sites [24]. The headache began to improve and frequency of attacks diminished over several weeks until the mean pain VAS score was 2-3, although the patient still required lamotrigine 900 mg/d and gabapentin 1,600 mg/d. As we have experience in treating trigeminal neuralgia with botulinum toxin A [25], we chose this approach for this child, administering a total dose of 70 U around the orbit, temporal area, and upper gum, a larger dose than that previously reported [24]. Our patient began to experience an improvement in symptoms after 4 days; he was pain-free by 7 days and had stopped all drugs by 11 days, a more rapid response than expected. Consequently, we recommend that the dose of botulinum toxin A can be titrated against response and that more can be administered if pain is not completely relieved. Once the patient is pain-free, then their analgesics can be slowly weaned and stopped if possible. Our patient has experienced no further symptoms or side effects over the 17 months of follow-up. The botulinum toxin A option provides a much less invasive option than the surgical interventions that have been described earlier in the paper. We recommend that the use of botulinum toxin A can be considered as a first-line treatment for children (≥12 years old) suffering from a first episode of SUNCT syndrome.
